# Excitability of the Ipsilateral Primary Motor Cortex During Unilateral Goal-Directed Movement

**DOI:** 10.3389/fnhum.2021.617146

**Published:** 2021-02-17

**Authors:** Takuya Matsumoto, Tatsunori Watanabe, Takayuki Kuwabara, Keisuke Yunoki, Xiaoxiao Chen, Nami Kubo, Hikari Kirimoto

**Affiliations:** ^1^Department of Sensorimotor Neuroscience, Graduate School of Biomedical and Health Sciences, Hiroshima University, Hiroshima, Japan; ^2^Research Fellow of Japan Society for the Promotion of Science, Tokyo, Japan

**Keywords:** transcranial magnetic stimulation, intracortical inhibition, ipsilateral primary motor cortex, laterality, goal-directed movement

## Abstract

**Introduction:**

Previous transcranial magnetic stimulation (TMS) studies have revealed that the activity of the primary motor cortex ipsilateral to an active hand (ipsi-M1) plays an important role in motor control. The aim of this study was to investigate whether the ipsi-M1 excitability would be influenced by goal-directed movement and laterality during unilateral finger movements.

**Method:**

Ten healthy right-handed subjects performed four finger tapping tasks with the index finger: (1) simple tapping (Tap) task, (2) Real-word task, (3) Pseudoword task, and (4) Visually guided tapping (VT) task. In the Tap task, the subject performed self-paced simple tapping on a touch screen. In the real-word task, the subject tapped letters displayed on the screen one by one to create a Real-word (e.g., apple). Because the action had a specific purpose (i.e., creating a word), this task was considered to be goal-directed as compared to the Tap task. In the Pseudoword task, the subject tapped the letters to create a pseudoword (e.g., gdiok) in the same manner as in the Real-word task; however, the word was less meaningful. In the VT task, the subject was required to touch a series of illuminated buttons. This task was considered to be less goal-directed than the Pseudoword task. The tasks were performed with the right and left hand, and a rest condition was added as control. Single- and paired-pulse TMS were applied to the ipsi-M1 to measure corticospinal excitability and short- and long-interval intracortical inhibition (SICI and LICI) in the resting first dorsal interosseous (FDI) muscle.

**Results:**

We found the smaller SICI in the ipsi-M1 during the VT task compared with the resting condition. Further, both SICI and LICI were smaller in the right than in the left M1, regardless of the task conditions.

**Discussion:**

We found that SICI in the ipsi-M1 is smaller during visual illumination-guided finger movement than during the resting condition. Our finding provides basic data for designing a rehabilitation program that modulates the M1 ipsilateral to the moving limb, for example, for post-stroke patients with severe hemiparesis.

## Introduction

Transcranial magnetic stimulation (TMS) is one of the tools for the non-invasive examination of the excitability of human primary motor cortex (M1). Previous TMS studies have revealed that the activity of the ipsilateral to the active hand (ipsi-M1) plays an important role in motor control (Tinazzi and Zanette, 1998; Buetefisch et al., 2014; Reid and Serrien, 2014). For example, when TMS is applied over the ipsi-M1 to elicit motor evoked potentials (MEPs) in a resting hand, their amplitudes are larger during complex than during simple movements (Tinazzi and Zanette, 1998; Morishita et al., 2011, 2012). Meanwhile, MEP amplitude was found to be larger during the observation of grasping than during the observation of simple arm movement (Fadiga et al., 1995). Furthermore, observation of actual grasp was demonstrated to induce larger MEPs than observation of pantomimed (or meaningless) grasp, which was defined as an intransitive movement not associated with a particular goal (Enticott et al., 2010). Despite these findings indicating that the corticospinal excitability can be modulated by the goal-directedness of a movement, its effect on the ipsi-M1 excitability has not been elucidated fully.

In addition, laterality has also been shown to affect the excitability of M1. For instance, in right-handed individuals, the threshold for muscle activation was lower in the right arm compared with the left arm (Triggs et al., 1994). Also, intracortical inhibition has been found to be stronger in the left than in the right M1 during a resting state (Civardi et al., 2000; Hammond et al., 2004; Hammond and Garvey, 2006). Furthermore, the excitability of the ipsi-M1 was larger for the tasks performed with the non-dominant left hand than for those executed with the dominant right hand (Ziemann and Hallett, 2001; Ghacibeh et al., 2007; Morishita et al., 2011; Reid and Serrien, 2014). Therefore, it is necessary to examine whether laterality influences the effect of goal-directedness on ipsi-M1 activity.

Accordingly, in this study we tested the hypothesis that ipsi-M1 excitability and intracortical inhibitory circuits would be influenced by goal-directedness and laterality during unilateral finger movements. If goal-directed movements can enhance the activity of the ipsi-M1, these movements may be applicable to stroke rehabilitation, since increased activity of the ipsilesional M1 is crucial for successful rehabilitation in hemiparetic post-stroke patients (Carey et al., 2005; Yamada et al., 2013).

## Materials and Methods

### Subjects

Ten healthy volunteers (7 males and 3 females, 21.4 ± 1.26 years, mean ± SD) participated in this study. All participants provided written informed consent prior to the experiment, which was conducted in accordance with the principles of the Declaration of Helsinki. All participants were right-hand dominant (Laterality Quotient 99.0 ± 3.16, mean ± SD) according to the Edinburgh Handedness Inventory (Oldfield, 1971). The protocol was approved by the Ethics Committee of Niigata University of Health and Welfare.

### Experimental Procedure

The subject was seated with their arms resting comfortably on a table and was asked to perform four finger tapping tasks with the dominant and non-dominant index fingers using a touch screen (FDX10001T, EIZO, Japan), which was placed on the table.

### Unilateral Finger Tapping Task

**FIGURE 1:**
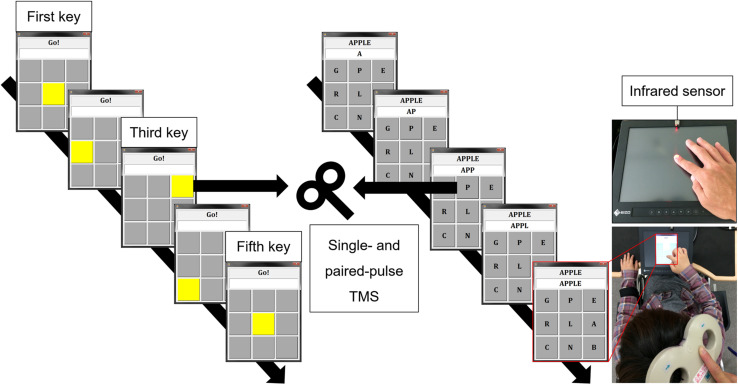
Experimental setting. Tap task **(upper right)**: self-paced finger tapping on a touch screen. VT task **(left)**: pressing the illuminated button (yellow key) one by one. Real-word task **(middle)**: creating a real word (e.g., apple) by tapping letters one by one. Infrared sensor and touch on screen (third key) were used as TMS trigger for the Tap task and the other tasks, respectively. TMS: transcranial magnetic stimulation, VT: visually guided finger tapping task.

(1) Simple tapping (Tap) task: The subject performed self-paced simple tapping (five taps) on a touch screen.
(2) Real-word task: A real five-letter word (e.g., apple) and nine letters arranged in a 3 × 3 matrix were displayed on the touch screen, and the subject tapped the letters on the screen one by one to create the displayed word. We selected simple English words of five letters (taught in junior high school in Japan) to exclude possible differences in letter search time and tapping speed. Because there was a movement goal (i.e., creating a word), this task was considered to be goal-directed, as compared to the Tap task (Gordon et al., 1998).
(3) Pseudoword task: The subject tapped the letters to create a pseudoword (e.g., gdiok) in the same manner as in the Real-word task, i.e., the subject tapped the letters on the screen to create the displayed pseudoword (not on a whim). Although creating a word (Real-word task) was considered to be more goal-directed than simple tapping (Tap task), the number of muscles involved in these tapping tasks was different as the Real-word task involved wrist movements. In addition, visual stimuli were used in the Real-word task. To control these factors, the Pseudoword task was included: the subject produced approximately the same amount of movement as in the Real-word task; however, the word was less goal-directed.
(4) Visually guided finger tapping (VT) task: Nine buttons (3 × 3 matrix) on the touch screen turned yellow one by one, and the subject was required to touch the illuminated button. This task to simply follow the illumination was considered to be less goal-directed than the Pseudoword task. Figure 1 and Table 1 show the experimental settings and the characteristics of each task, respectively. Before starting the experimental session, we explained the procedure of the finger tapping tasks to the subject and asked her/him to practice the tasks. The subject practiced each motor task for approximately 5 min, respectively. The examiner confirmed that the subject was able to perform the word creation task and the visually guided finger tapping task without missing a tap. During the tapping tasks, except for the Tap task, the keys to be tapped (target keys) were randomly presented on the screen. Experimental tasks with the dominant and non-dominant hands were tested in separate sessions on different days, and the order of these tasks were randomized among the subjects. The tasks were spaced by resting periods of at least 1 min.

**TABLE 1:** Characteristics of unilateral finger tapping tasks.

	Finger movement	Wrist movement	Goal-directedness
Resting condition	–	–	–
Tap task	+	–	±
VT task	+	+	+
Pseudoword task	+	+	++
Real-word task	+	+	+++

### Electromyography Recording

Surface electromyography (EMG) was recorded from the first dorsal interosseous (FDI) muscles of the dominant right hand and the non-dominant left hand using disposable silver-silver chloride surface electrodes. The recording and reference electrodes were placed over the muscle belly and tendon, respectively. The EMG signals were amplified (×100; DL-140, 4 assist, Japan), band-pass filtered between 5 and 1,000 Hz, digitized at 4k Hz using an analog-to-digital converter (PowerLab, AD Instruments, Australia), and stored in a personal computer for off-line analysis (LabChart 7, AD Instruments, Australia). Prior to the experimental sessions, we examined the maximal voluntary contraction (MVC) of the FDI muscle. The subject was asked to gradually increase the force from zero to maximum over 3 s and to maintain maximal force for 3 s by abducting the index finger. The subjects received visual feedback and verbal encouragement during MVC. The subject performed three trials with resting for at least 90 s between trials (Maluf et al., 2005; Kirimoto et al., 2014).

### Transcranial Magnetic Stimulation Measurement

Transcranial Magnetic Stimulation was delivered using a figure-of-eight coil (external loop diameter of 95 mm) connected to two stimulators (Magstim 200, Magstim, United Kingdom). The coil was manually fixed tangentially to the sculp with the handle pointing in a posterolateral direction at an angle of 45° from the midsagittal line. The subject wore a swimming cap, and the outer edge of the coil was marked with a pen so that the position of the coil would not change during the experiment. The site where weak TMS consistently evoked the largest MEP in the FDI muscle was determined as the motor hotspot. The resting motor threshold (rMT) was defined according to international guidelines (Rossini et al., 2015). RMT was defined as the lowest stimulus intensity was required to elicit MEP amplitude (>50 μV) in about 50% of 10 consecutive stimuli. During the experimental tasks (four finger tapping tasks) and a rest control condition, single- and paired-pulse TMS were applied to the M1 ipsilateral to the hand performing the task to measure corticospinal excitability and intracortical inhibition in the resting FDI muscle. Specifically, when the subject performed the tapping task with the dominant right hand (*active*), TMS was applied to the right (ipsilateral) M1 and MEPs were recorded from the left (*resting*) FDI muscle, and when the subject performed the tapping task with the non-dominant left hand (*active*), TMS was applied to the left (ipsilateral) M1 and MEPs were recorded from the right (*resting*) FDI muscle. The intensity of single-pulse TMS to measure corticospinal excitability was set to elicit MEP with a peak-to-peak amplitude of about 1 mV during resting condition and fixed across conditions in each subject. Paired-pulse TMS is widely used to evaluate non-invasively human M1 excitability. Application of a subthreshold conditioning stimulus (CS) followed by a suprathreshold test stimulus (TS) after short interstimulus interval (ISI) of 1–5 ms reduces the test MEP amplitude (Kujirai et al., 1993; Hanajima et al., 1998). Moreover, a suprathreshold CS with a long ISI of 50–150 ms similarly reduces amplitude of test MEP (Valls-Sole et al., 1992; Wassermann et al., 1996). Due to difference in ISI, these inhibitory phenomena are known as short-interval intracortical inhibition (SICI) and long-interval intracortical inhibition (LICI), respectively. SICI is likely mediated by γ-aminobutyric acid (GABA) type A (GABA_A_) receptors, and LICI by GABA type B (GABA_B_) receptors (Ziemann et al., 1996a; Nakamura et al., 1997; McDonnell et al., 2006). Further, they are thought to be of cortical origin (Nakamura et al., 1997). For both the SICI and LICI, the intensity of CS was adjusted to obtain a conditioned MEP amplitude of about 50% of the unconditioned MEP at rest to avoid a floor effect (Cirillo et al., 2011; Uehara et al., 2013b). The intensity of TS was set to elicit MEP with a peak-to-peak amplitude of about 1 mV during resting condition and motor tasks, respectively. The detail of TMS intensity is summarized in Table 2. ISI between CS and TS was 3 and 100 ms for SICI and LICI, respectively (Hanajima et al., 1998; Sanger et al., 2001). Single- and paired-pulse TMS were delivered randomly in the same session using a pulse stimulator (Random double-pulse system, 4 assist, Japan). The infrared sensor (FS-N11MN, Keyence Corporation, Japan) and the touch screen (third key press) were used as TMS trigger for the Tap task and the other tasks (Figure 1). TMS was applied once every five taps, and the subject tapped a total of 180 times until 36 MEPs (12 for each) were recorded in the *resting* FDI. Each task was divided into two sessions (90 taps for each) to avoid fatigue. Letter searching time may differ when the subject taps first or second key press, and tapping speed may vary when the subject executes the fourth and fifth key presses. Therefore, we used the third key press as TMS trigger to avoid these factors.

**TABLE 2:** Summary of the TMS intensity in each condition (mean ± SD, % of maximal stimulator output: %MSO).

	Single-pulse TMS		CS				TS	
			
	Non-dominant M1	Dominant M1	Non-dominant M1		Dominant M1		Non-dominant M1	Dominant M1
						
			SICI	LICI	SICI	LICI	TS	TS
Resting condition							56.8 ± 8.83	52.2 ± 7.38
Tap task							55.2 ± 9.31	52.3 ± 7.30
VT task	56.8 ± 8.83	52.2 ± 7.38	34.6 ± 5.15	53.7 ± 8.53	31.8 ± 6.14	49.7 ± 6.90	55.4 ± 8.25	52.2 ± 7.38
Pseudoword task							55.4 ± 8.25	51.7 ± 7.39
Real-word task							55.6 ± 8.40	52.0 ± 7.56

### Data and Statistical Analysis

Electromyography from the *active* FDI muscle (tapping hand) was rectified and normalized to the MVC value (% MVC). We then calculated the mean EMG activity in *active* and *resting* FDI muscles during a period of 100 ms just prior to the TMS pulse. In paired-pulse TMS, the mean EMG activity in both FDI muscles was calculated during a period of 100 ms just prior to the CS. We also calculated the peak-to-peak amplitude of MEP. SICI and LICI were expressed as the ratio of the conditioned MEP amplitude to the unconditioned MEP amplitude. A MEP ratio less than 1 indicated inhibition, whereas a MEP ratio greater than 1 indicated facilitation. All data were expressed as mean ± SEM. Two-way repeated-measures analysis of variance (ANOVA) was performed to examine the effects of laterality (dominant and non-dominant hands) and to evaluate the impact of the different conditions (Resting condition, Tap task, VT task, Pseudoword task, and Real-word task). The sphericity of the data was tested by the Mauchly’s test, and the Greenhouse-Geisser corrected significance values were tested when sphericity was not met. Bonferroni’s correction for multiple comparisons was used for post hoc analysis. A value of *p* < 0.05 was considered statically significant for all analyses. The effect size for each ANOVA was calculated using eta squared (η^2^) (Cohen, 1988).

## Results

Table 3 shows the amplitude of EMG activity in the *active* FDI muscle during the unilateral finger tapping tasks. The EMG activity was around 20% MVC for all the tasks, and there was no significant difference between them. Table 4 shows the amplitude of EMG activity in the *resting* FDI muscle during the resting condition and the motor tasks. Two-way repeated-measures ANOVA on the amplitude of EMG activity in the *resting* FDI muscle for SICI showed a significant main effect of laterality (Laterality; *F* (1,90) = 9.66, *p* = 0.003, η^2^ = 0.090, condition; *F* (4,90) = 1.70, *p* = 0.35, η^2^ = 0.042, interaction; *F* (4,90) = 2.06, *p* = 0.11, η^2^ = 0.031). Similarly, LICI showed significant main effect of laterality (Laterality; *F* (1,90) = 13.36, *p* = 0.001, η^2^ = 0.117, condition; *F* (4,90) = 0.98, *p* = 0.42, η^2^ = 0.034, interaction; *F* (4,90) = 1.77, *p* = 0.14, η^2^ = 0.062).

**TABLE 3:** Amplitude of EMG activity in the *active* FDI muscle (tapping hand) during unilateral finger tapping tasks (mean ± SEM, %MVC).

	Non-dominant hand				Dominant hand			
		
	Tap task	VT task	Pseudoword task	Real-word task	Tap task	VT task	Pseudoword task	Real-word task
Single-pulse TMS	29.8 ± 5.75	19.8 ± 2.58	21.4 ± 2.98	15.7 ± 2.75	21.9 ± 3.60	21.7 ± 3.79	20.1 ± 3.75	18.8 ± 3.51
SICI	24.3 ± 4.73	18.8 ± 3.35	19.2 ± 2.14	15.3 ± 2.21	24.7 ± 3.83	19.2 ± 2.83	20.8 ± 3.48	21.1 ± 4.24
LICI	27.2 ± 5.67	23.3 ± 3.36	16.5 ± 2.67	17.4 ± 3.15	23.0 ± 4.24	22.0 ± 3.46	18.0 ± 3.44	18.1 ± 3.59

**TABLE 4:** Amplitude of EMG activity in the *resting* FDI muscle during rest condition and unilateral finger tapping tasks (mean ± SEM, μV).

	Non-dominant hand					Dominant hand				
		
	Resting condition	Tap task	VT task	Pseudoword task	Real-word task	Resting condition	Tap task	VT task	Pseudoword task	Real-word task
Single-pulse TMS	4.56 ± 0.32	5.16 ± 0.66	4.64 ± 0.34	5.57 ± 0.88	5.24 ± 0.59	3.35 ± 0.26	4.71 ± 0.51	4.69 ± 0.59	4.23 ± 0.44	4.30 ± 0.66
SICI	4.49 ± 0.34	4.88 ± 0.45	4.78 ± 0.29	5.59 ± 0.51	4.52 ± 0.43	3.67 ± 0.42	4.52 ± 0.36	4.21 ± 0.37	3.98 ± 0.25	4.05 ± 0.41
LICI	4.76 ± 0.33	4.81 ± 0.54	4.64 ± 0.41	6.10 ± 0.80	4.46 ± 0.30	3.48 ± 0.31	4.22 ± 0.39	3.85 ± 0.34	3.76 ± 0.34	4.31 ± 0.45

**FIGURE 2:**
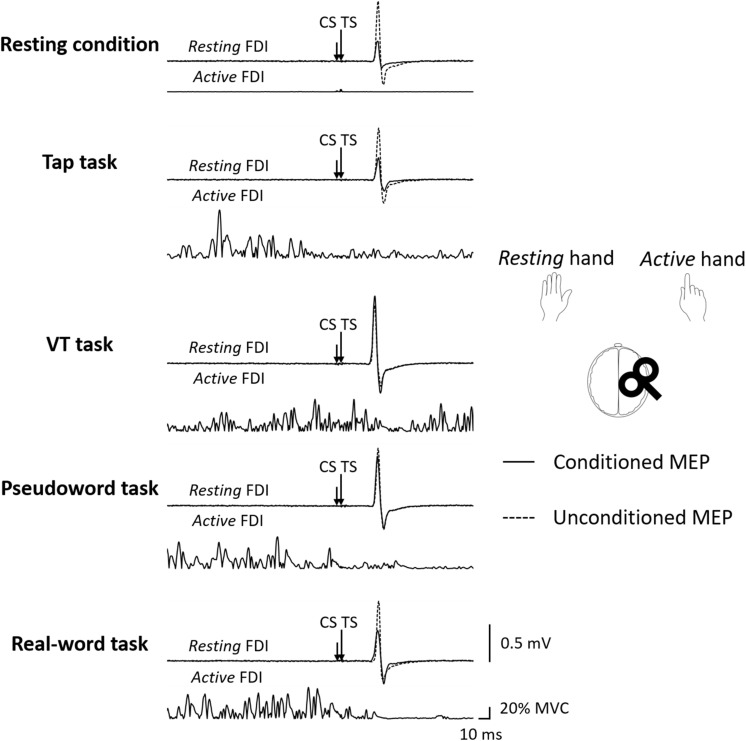
The representative MEP waveforms evoked by paired-pulse TMS (ISI 3 ms) during each condition. The subject performed the unilateral finger tapping tasks with the dominant right hand (*active*), TMS was delivered to the ipsilateral (right) M1, and MEPs were recorded from left (*resting*) FDI muscle. CS: conditioning stimulus, FDI: first dorsal interosseous, ISI: interstimulus interval, MEP: motor evoked potential, TMS: transcranial magnetic stimulation, TS: test stimulus, VT: visually guided tapping task.

**FIGURE 3:**
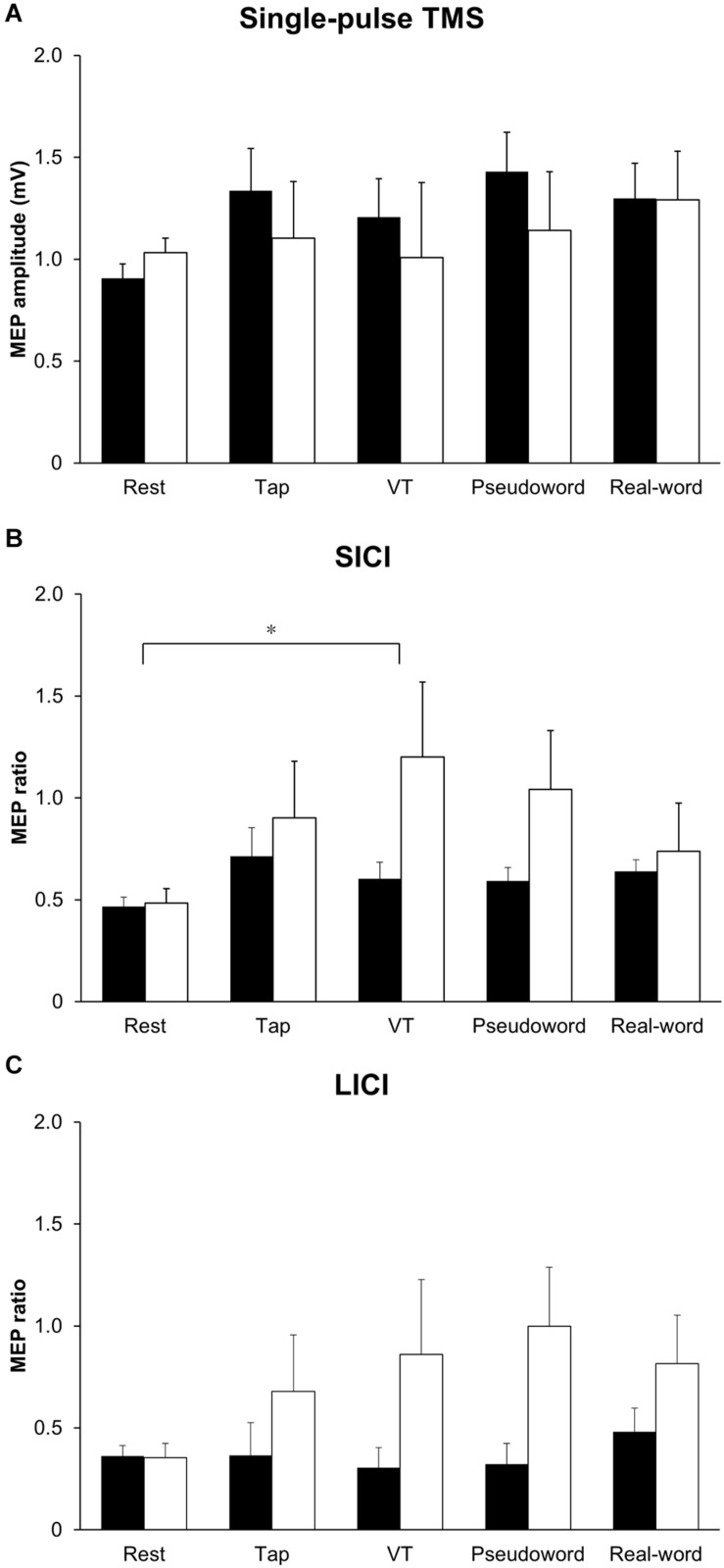
Group data (*n* = 10) of **(A)** MEP amplitude in the *resting* FDI muscle following single-pulse TMS, **(B)** MEP ratio for SICI, and **(C)** MEP ratio for LICI in each condition. A MEP ratio less than 1 indicates inhibition, and a MEP ratio greater than 1 indicates facilitation. The closed bars represent the results of the non-dominant hand performance and the corresponding resting condition, and the opened bars represent the results of the dominant hand performance and the corresponding resting condition. SICI was significantly smaller in the VT task than in the resting condition (*p < 0.05) **(B)**. FDI: first dorsal interosseous, LICI: long-interval intracortical-inhibition, MEP: motor evoked potential, SICI: short-interval intracortical inhibition, TMS: transcranial magnetic stimulation. VT: visually guided tapping task.

Figure 2 shows the representative MEPs. Figure 3A shows MEP amplitude in the *resting* FDI muscle following single-pulse TMS. Two-way repeated-measures ANOVA on single-pulse MEP amplitude revealed no main effect or interaction between laterality and condition (laterality; *F* (1,90) = 0.71, *p* = 0.40, η^2^ = 0.007, condition; *F* (4,90) = 0.74, *p* = 0.57, η^2^ = 0.031, interaction; *F* (4,90) = 0.30, *p* = 0.88, η^2^ = 0.013).

Figures 3B,C show MEP ratio for SICI and LICI. Unconditioned MEP amplitude in the *resting* FDI muscle following single-pulse TS during unilateral finger tapping tasks performed with the dominant and non-dominant hands were comparable (non-dominant hand: Tap task 0.81 ± 0.16 mV; VT task 0.96 ± 0.13 mV; Pseudoword task 1.06 ± 0.11 mV; Real-word task 1.10 ± 0.15 mV. Dominant hand: Tap task 1.20 ± 0.23 mV; VT task 0.97 ± 0.23 mV; Pseudoword task 1.07 ± 0.28 mV; Real-word task 1.09 ± 0.21 mV). Two-way repeated-measures ANOVA on MEP ratio for SICI showed significant main effects of laterality (*F* (1,90) = 8.94, *p* = 0.004, η^2^ = 0.078) and condition (*F* (4,90) = 2.68, *p* = 0.04, η^2^ = 0.092). The laterality effect indicated that SICI was smaller in the right than in the left M1. Post hoc analysis revealed a significantly smaller SICI during the VT task than during the resting condition (*p* < 0.05). No interaction between laterality and condition was found (*F* (4,90) = 1.46, *p* = 0.22, η^2^ = 0.051). Two-way repeated-measures ANOVA on MEP ratio for LICI showed a significant main effect of laterality (*F* (1,90) = 7.89, *p* = 0.01, η^2^ = 0.066), but there was no main effect of condition (*F* (4,90) = 0.68, *p* = 0.61, η^2^ = 0.026) or interaction (*F* (4,90) = 0.78, *p* = 0.54, η^2^ = 0.033). The main effect of laterality indicated that LICI was smaller in the right than in the left M1.

## Discussion

We investigated whether excitability of the M1 ipsilateral to the active hand would be influenced by the goal-directedness of the movement and laterality during unilateral finger movements using motor tasks whose goal-directedness was systematically adjusted. As a result, our findings indicated that (1) unexpectedly performing a goal-directed movement does not necessarily result in a greater reduction of intracortical inhibitory circuits in the ipsi-M1, (2) SICI in the ipsi-M1 can be smaller during visual illumination-guided finger movement as compared to the resting condition, and (3) intracortical inhibitory circuits in the ipsi-M1 is smaller in the right than in the left M1.

### Effect of Goal-Directed Movement on the Ipsi-M1 Activity

Tinazzi and Zanette (1998) examined the ipsi-M1 excitability in different finger opposition tasks and found greater ipsi-M1 excitability during sequential finger opposition than during simple opposition with the third finger and thumb. In addition, Morishita et al. (2011) compared the excitability of the ipsi-M1 between fine motor (chopsticks manipulation) and pseudo-fine motor (repetitive grasping with the thumb and index and middle fingers) tasks, and revealed that the excitability of the ipsi-M1 was larger during the fine motor task. These results indicate that the excitability of the ipsi-M1 is larger during complex movements than during simple movements. Meanwhile, the corticospinal excitability was found to be larger during the observation of the goal-directed movement than during the observation of meaningless movements (Enticott et al., 2010). From this evidence, we assumed that the ipsi-M1 excitability would increase as the task becomes more goal-directed (i.e., tapping letters on a screen one by one to create a word). However, the corticospinal excitability and intracortical inhibition were not influenced by the goal-directed task in this study, and alternatively we found a smaller SICI in the ipsi-M1 during the visually guided finger tapping task compared with the resting condition. These results suggest that simple visual guidance rather than goal-directed movement is key to the modulation of SICI in the ipsi-M1.

Several studies have examined M1 excitability and SICI during cognitive tasks. For instance, in Stop Signal and Go/No-Go tasks, SICI in the contralateral M1 was demonstrated to be greater during the stop and No-Go trials than during the go trial (Sohn et al., 2002; Coxon et al., 2006; Lindberg et al., 2016). These results indicate that SICI is involved in the selection and inhibition of voluntary movements. In the present study, subjects were requested to select letters in order to create a word in the Real-word and Pseudoword tasks, and this selection requirement seemed to be much lower in the visually guided motor task. We speculate that this characteristic of the VT task affected SICI in the ipsi-M1.

Besides those cognitive task studies, several studies have examined the modulation of SICI using triple-pulse TMS. For instance, CS applied over the premotor area before the CS for SICI was found to reduce the test MEP amplitude (Mochizuki et al., 2004). Moreover, SICI was found to be reduced by the interhemispheric inhibition (IHI) that occurs between the bilateral M1s and also by the cerebellar inhibitory input (Daskalakis et al., 2002, 2004). Since the cerebellar-cortical circuit including premotor area can play an important role in externally triggered movements (Taniwaki et al., 2006), activities of the cerebellar or premotor areas may have been involved in the reduction of SICI in the ipsi-M1 during the VT task.

One possible explanation for the insignificant effect of goal-directed task could be that the influence was more evident in brain areas and networks that cannot be evaluated by single- or paired-pulse TMS. Previous brain imaging studies demonstrated that the supplementary motor area (Shibasaki et al., 1993) and the premotor area (Kawashima et al., 1998; Verstynen and Ivry, 2011) play important roles in the execution of complex finger movements. In addition, the interhemispheric connection between the bilateral somatosensory cortices and the corticocortical connections between the sensorimotor and premotor areas are involved in the control of limb movements (Bundy and Leuthardt, 2019). Thus, the connectivity between the M1 and high-order cortical regions possibly involved in goal-directed movements (Iacoboni et al., 2005) should be investigated using functional brain imaging and electroencephalography in future studies.

### Laterality of Intracortical Inhibitory Circuits Within the Ipsi-M1

We found that SICI and LICI in the ipsi-M1 were smaller in the right than in the left M1. It has been reported that SICI and LICI are stronger in the left than in the right M1 during the resting state in right-handed individuals (Civardi et al., 2000; Hammond et al., 2004; Hammond and Garvey, 2006). On the other hand, results regarding the SICI in the ipsi-M1 during movements in terms of laterality have been inconsistent. Some studies showed reduced SICI only in the right M1 (Hinder et al., 2010; van den Berg et al., 2011), while the others showed reduced SICI only in the left M1 (Morishita et al., 2011). Since not enough research has been conducted on LICI, cross-study comparisons cannot be made in this regard. Nevertheless, our results seem to support the previous findings that intracortical inhibitory circuits are stronger in the left than in the right M1. We speculate that more complex intracortical connections in the left than in the right M1 (Amunts et al., 1996), greater dexterity in dominant than non-dominant hand (Hammond et al., 2004), and stronger IHI from left to right M1 (Netz et al., 1995) contributed to the laterality of intracortical inhibitory circuits observed in the present study.

### Corticospinal Excitability

There was no difference in MEP amplitude elicited by single-pulse TMS between tasks. This indicates that corticospinal excitability was not influenced by goal-directed movement, and that the corticospinal excitability did not change in the VT task despite the finding of reduction in SICI. Corticospinal excitability depends on the balance between excitatory and inhibitory neural systems within the M1, which are distinct from the corticospinal pathway. These neural systems are thought to not only modulate corticospinal excitability but also interact with each other within the M1 (Sanger et al., 2001; Reis et al., 2008; Ni et al., 2011). Di Lazzaro et al. (2002) and Fierro et al. (2010) used repetitive TMS to examine corticospinal excitability and intracortical inhibitory circuits at rest. Meanwhile, Smyth et al. (2010) and Quinn et al. (2018) used motor tasks to measure effect of motor learning on corticospinal excitability and intracortical inhibitory circuits. In this relation, some studies have shown a decrease in SICI and no change in corticospinal excitability (Di Lazzaro et al., 2002; Smyth et al., 2010), while the others showed a decrease in both SICI and corticospinal excitability (Fierro et al., 2010; Quinn et al., 2018). Quinn et al. (2018) investigated corticospinal excitability and SICI in the forearm flexor and extensor muscles during a visuomotor task, and found that the corticospinal excitability was reduced in both the forearm flexor and extensor muscles, while SICI was reduced only in the forearm extensor muscle. It is known that inhibitory and excitatory circuits can act independently in M1 (Ziemann et al., 1996b; Liepert et al., 1998); thus, changes in SICI may not be directly related to changes in corticospinal excitability. Our findings are in line with these observations. Additionally, the effect of motor task on the ipsi-M1 activity may depend on the type of motor task. While we used a phasic tapping task, previous TMS studies that demonstrated an increase in the ipsi-M1 excitability and a decrease in SICI used a static contraction task (Muellbacher et al., 2000; Liang et al., 2008, 2014). Furthermore, Liepert et al. (2001) revealed that the ipsi-M1 excitability was larger during static contraction than phasic contraction. Therefore, the discrepancy with previous studies may be due to differences in the task/contraction type.

### Long-Interval Intracortical Inhibition

Similar to the corticospinal excitability, LICI was not different between the tasks. The only study that has investigated LICI in the ipsi-M1 during unilateral finger movement was the research conducted by Uehara et al. (2013b). They reported reduced LICI in the ipsi-M1 during repetitive finger abduction paced according to auditory cues (Uehara et al., 2013b). In addition, LICI in the M1 contralateral to active hand was found to be smaller during precision grip than during index finger abduction, and the synergic movement of the thumb and index finger along with their afferent inputs are thought to contribute to the reduction of LICI (Kouchtir-Devanne et al., 2012; Caux-Dedeystere et al., 2014). These findings possibly suggest that LICI is involved in movements requiring force control rather than in goal-directed movements.

There is a confounding factor that could affect the result of LICI. Specifically, the timing of TS for LICI was different from that of SICI. TS for single-pulse TMS and SICI was delivered immediately after the key tap, whereas TS for LICI was delivered 100 ms after the key tap. Therefore, it is possible that SICI and LICI were assessed during different cognitive and motor processes. In a study by Uehara et al. (2013a), TMS was delivered over the ipsi-M1 using a different interval from EMG onset to TMS (0–500 ms), and ipsi-M1 excitability was found to be independent of this interval when low intensity contraction (30% MVC) was used. Because the motor tasks used in this study were performed at a low intensity (approximately 20% MVC) and timing of TS was within 500 ms, it is unlikely that the timing of TS affected the excitability of the ipsi-M1.

### Potential Application to Rehabilitation

Although patients with mild to moderate hemiparesis can perform exercises with the affected arm to some degree and hence have a relatively favorable clinical prognosis (Kwakkel et al., 2003), those with severe hemiparesis have poorer prognosis because of limited voluntary control (Kwakkel et al., 2003). As increased activity of the ipsilesional M1 is a key to successful rehabilitation in hemiparetic post-stroke patients (Carey et al., 2005; Yamada et al., 2013), motor exercise of the unaffected limb to enhance ipsilesional M1 activity may become one of the means to facilitate motor recovery in post-stroke patients with severe symptoms. From this perspective, our findings suggest a potential use of visual guidance to enhance the ipsilesional M1 activity. More thorough investigations will be necessary, however, to confirm this interesting possibility.

## Limitations

This study has some limitations. First, there was a significant difference in EMG activity in the *resting* FDI muscle between the dominant and non-dominant hands (Table 4). However, these EMG activities were very small (less than 10 μV) and Cavanagh and Komi (1979) defined muscle activity as above 30 μV. Hence, background EMG was not a confounding factor for MEP results. Second, we recorded EMG activity only from the FDI muscle. The motor tasks, except for the Tap task, involved the movement of multiple joints, including the fingers and wrist. Nevertheless, MEP amplitude did not differ between the Tap task and the other motor tasks. Hence, it is unlikely that the multiple joints movement affected MEP amplitude. Third, we did not assess IHI between hemispheres. As the interhemispheric interaction can be modulated by goal-directed movements, it should be examined in a future study. Fourth, we measured SICI only with ISI of 3 ms and did not assess short-interval intracortical facilitation. A thorough examination of SICI with an ISI of 1–4 ms and short-interval intracortical facilitation may provide more detailed mechanisms. Finally, our sample size was small, and although we consider that the effect size was medium (Cohen, 1988), increasing the number of subjects may allow for a better understanding of the differences found in this study.

## Conclusion

In conclusion, unexpectedly we found that SICI in the ipsi-M1 is smaller during visual illumination-guided finger movement than during the resting condition. Less selection requirements during the visually guided movements could be the underlying reason. The laterality of intracortical inhibitory circuits in the ipsi-M1 could be associated with hemispheric asymmetry. Our findings provide basic data for the development of a rehabilitation program that modulates the M1 ipsilateral to the moving limb, which could be used, for example, for post-stroke patients with severe hemiparesis.

## Data Availability Statement

The original contributions presented in the study are included in the article/supplementary material, further inquiries can be directed to the corresponding author/s.

## Ethics Statement

The studies involving human participants were reviewed and approved by the Ethics Committee of Niigata University of Health and Welfare. The patients/participants provided their written informed consent to participate in this study.

## Author Contributions

HK and TM designed the study. TM and TK performed the experiment. TM analyzed the data and wrote the initial draft of the manuscript. KY, XC, and NK assisted in the preparation of the manuscript. HK and TW edited and revised the manuscript. All authors approved the final version of the manuscript, and agreed to be accountable for all aspect of the work in ensuring that questions related to the accuracy or integrity of any part of the work are appropriately investigated and resolved.

## Conflict of Interest

The authors declare that the research was conducted in the absence of any commercial or financial relationships that could be construed as a potential conflict of interest.

## References

[B1] Amunts K. Schlaug G. Schleicher A. Steinmetz H. Dabringhaus A. Roland P. E. (1996). Asymmetry in the human motor cortex and handedness. *Neuroimage* 4(3 Pt 1) 216–222. 10.1006/nimg.1996.0073 9345512

[B2] Buetefisch C. M. Revill K. P. Shuster L. Hines B. Parsons M. (2014). Motor demand-dependent activation of ipsilateral motor cortex. *J. Neurophysiol.* 112 999–1009. 10.1152/jn.00110.2014 24848477 PMC4122744

[B3] Bundy D. T. Leuthardt E. C. (2019). The cortical physiology of ipsilateral limb movements. *Trends Neurosci.* 42 825–839. 10.1016/j.tins.2019.08.008 31514976 PMC6825896

[B4] Carey L. M. Abbott D. F. Egan G. F. Bernhardt J. Donnan G. A. (2005). Motor impairment and recovery in the upper limb after stroke: behavioral and neuroanatomical correlates. *Stroke* 36 625–629. 10.1161/01.STR.0000155720.47711.83 15677574

[B5] Caux-Dedeystere A. Rambour M. Duhamel A. Cassim F. Derambure P. Devanne H. (2014). Task-dependent changes in late inhibitory and disinhibitory actions within the primary motor cortex in humans. *Eur. J. Neurosci.* 39 1485–1490. 10.1111/ejn.12505 24517419

[B6] Cavanagh P. R. Komi P. V. (1979). Electromechanical delay in human skeletal muscle under concentric and eccentric contractions. *Eur. J. Appl. Physiol. Occup. Physiol.* 42 159–163. 10.1007/BF00431022 527577

[B7] Cirillo J. Todd G. Semmler J. G. (2011). Corticomotor excitability and plasticity following complex visuomotor training in young and old adults. *Eur. J. Neurosci.* 34 1847–1856. 10.1111/j.1460-9568.2011.07870.x 22004476

[B8] Civardi C. Cavalli A. Naldi P. Varrasi C. Cantello R. (2000). Hemispheric asymmetries of cortico-cortical connections in human hand motor areas. *Clin. Neurophysiol.* 111 624–629. 10.1016/s1388-2457(99)00301-6 10727913

[B9] Cohen J. (1988). *Statistical Power Analysis for the Behavioral Sciences*, 2nd Edn. New York, NY: Routledge Academic.

[B10] Coxon J. P. Stinear C. M. Byblow W. D. (2006). Intracortical inhibition during volitional inhibition of prepared action. *J. Neurophysiol.* 95 3371–3383. 10.1152/jn.01334.2005 16495356

[B11] Daskalakis Z. J. Christensen B. K. Fitzgerald P. B. Roshan L. Chen R. (2002). The mechanisms of interhemispheric inhibition in the human motor cortex. *J. Physiol.* 543(Pt 1) 317–326. 10.1113/jphysiol.2002.017673 12181302 PMC2290496

[B12] Daskalakis Z. J. Paradiso G. O. Christensen B. K. Fitzgerald P. B. Gunraj C. Chen R. (2004). Exploring the connectivity between the cerebellum and motor cortex in humans. *J. Physiol.* 557(Pt 2) 689–700. 10.1113/jphysiol.2003.059808 15047772 PMC1665103

[B13] Di Lazzaro V. Oliviero A. Mazzone P. Pilato F. Saturno E. Dileone M. (2002). Short-term reduction of intracortical inhibition in the human motor cortex induced by repetitive transcranial magnetic stimulation. *Exp. Brain Res.* 147 108–113. 10.1007/s00221-002-1223-5 12373375

[B14] Enticott P. G. Kennedy H. A. Bradshaw J. L. Rinehart N. J. Fitzgerald P. B. (2010). Understanding mirror neurons: evidence for enhanced corticospinal excitability during the observation of transitive but not intransitive hand gestures. *Neuropsychologia* 48 2675–2680. 10.1016/j.neuropsychologia.2010.05.014 20470809

[B15] Fadiga L. Fogassi L. Pavesi G. Rizzolatti G. (1995). Motor facilitation during action observation: a magnetic stimulation study. *J. Neurophysiol.* 73 2608–2611. 10.1152/jn.1995.73.6.2608 7666169

[B16] Fierro B. De Tommaso M. Giglia F. Giglia G. Palermo A. Brighina F. (2010). Repetitive transcranial magnetic stimulation (rTMS) of the dorsolateral prefrontal cortex (DLPFC) during capsaicin-induced pain: modulatory effects on motor cortex excitability. *Exp. Brain Res.* 203 31–38. 10.1007/s00221-010-2206-6 20232062

[B17] Ghacibeh G. A. Mirpuri R. Drago V. Jeong Y. Heilman K. M. Triggs W. J. (2007). Ipsilateral motor activation during unimanual and bimanual motor tasks. *Clin. Neurophysiol.* 118 325–332. 10.1016/j.clinph.2006.10.003 17095289

[B18] Gordon A. M. Lee J. H. Flament D. Ugurbil K. Ebner T. J. (1998). Functional magnetic resonance imaging of motor, sensory, and posterior parietal cortical areas during performance of sequential typing movements. *Exp. Brain Res.* 121 153–166. 10.1007/s002210050447 9696384

[B19] Hammond G. Faulkner D. Byrnes M. Mastaglia F. Thickbroom G. (2004). Transcranial magnetic stimulation reveals asymmetrical efficacy of intracortical circuits in primary motor cortex. *Exp. Brain Res.* 155 19–23. 10.1007/s00221-003-1696-x 15064880

[B20] Hammond G. R. Garvey C. A. (2006). Asymmetries of long-latency intracortical inhibition in motor cortex and handedness. *Exp. Brain Res.* 172 449–453. 10.1007/s00221-006-0349-2 16463150

[B21] Hanajima R. Ugawa Y. Terao Y. Sakai K. Furubayashi T. Machii K. (1998). Paired-pulse magnetic stimulation of the human motor cortex: differences among I waves. *J. Physiol. Lon.* 509 607–618. 10.1111/j.1469-7793.1998.607bn.x 9575308 PMC2230978

[B22] Hinder M. R. Schmidt M. W. Garry M. I. Summers J. J. (2010). The effect of ballistic thumb contractions on the excitability of the ipsilateral motor cortex. *Exp. Brain Res.* 201 229–238. 10.1007/s00221-009-2029-5 19826798

[B23] Iacoboni M. Molnar-Szakacs I. Gallese V. Buccino G. Mazziotta J. C. Rizzolatti G. (2005). Grasping the intentions of others with one’s own mirror neuron system. *PLoS Biol.* 3:e79. 10.1371/journal.pbio.0030079 15736981 PMC1044835

[B24] Kawashima R. Matsumura M. Sadato N. Naito E. Waki A. Nakamura S. (1998). Regional cerebral blood flow changes in human brain related to ipsilateral and contralateral complex hand movements–a PET study. *Eur. J. Neurosci.* 10 2254–2260. 10.1046/j.1460-9568.1998.00237.x 9749754

[B25] Kirimoto H. Tamaki H. Suzuki M. Matsumoto T. Sugawara K. Kojima S. (2014). Sensorimotor modulation differs with load type during constant finger force or position. *PLoS One* 9:e108058. 10.1371/journal.pone.0108058 25233353 PMC4169486

[B26] Kouchtir-Devanne N. Capaday C. Cassim F. Derambure P. Devanne H. (2012). Task-dependent changes of motor cortical network excitability during precision grip compared to isolated finger contraction. *J. Neurophysiol.* 107 1522–1529. 10.1152/jn.00786.2011 22157124

[B27] Kujirai T. Caramia M. D. Rothwell J. C. Day B. L. Thompson P. D. Ferbert A. (1993). Corticocortical inhibition in human motor cortex. *J. Physiol.* 471 501–519. 10.1113/jphysiol.1993.sp019912 8120818 PMC1143973

[B28] Kwakkel G. Kollen B. J. van der Grond J. Prevo A. J. (2003). Probability of regaining dexterity in the flaccid upper limb: impact of severity of paresis and time since onset in acute stroke. *Stroke* 34 2181–2186. 10.1161/01.STR.0000087172.16305.CD 12907818

[B29] Liang N. Funase K. Takahashi M. Matsukawa K. Kasai T. (2014). Unilateral imagined movement increases interhemispheric inhibition from the contralateral to ipsilateral motor cortex. *Exp. Brain Res.* 232 1823–1832. 10.1007/s00221-014-3874-4 24562411

[B30] Liang N. Murakami T. Funase K. Narita T. Kasai T. (2008). Further evidence for excitability changes in human primary motor cortex during ipsilateral voluntary contractions. *Neurosci. Lett.* 433 135–140. 10.1016/j.neulet.2007.12.058 18261851

[B31] Liepert J. Classen J. Cohen L. G. Hallett M. (1998). Task-dependent changes of intracortical inhibition. *Exp. Brain Res.* 118 421–426. 10.1007/s002210050296 9497149

[B32] Liepert J. Dettmers C. Terborg C. Weiller C. (2001). Inhibition of ipsilateral motor cortex during phasic generation of low force. *Clin. Neurophysiol.* 112 114–121. 10.1016/s1388-2457(00)00503-4 11137668

[B33] Lindberg P. G. Teremetz M. Charron S. Kebir O. Saby A. Bendjemaa N. (2016). Altered cortical processing of motor inhibition in schizophrenia. *Cortex* 85 1–12. 10.1016/j.cortex.2016.09.019 27770667

[B34] Maluf K. S. Shinohara M. Stephenson J. L. Enoka R. M. (2005). Muscle activation and time to task failure differ with load type and contraction intensity for a human hand muscle. *Exp. Brain Res.* 167 165–177. 10.1007/s00221-005-0017-y 16044306

[B35] McDonnell M. N. Orekhov Y. Ziemann U. (2006). The role of GABA(B) receptors in intracortical inhibition in the human motor cortex. *Exp. Brain Res.* 173 86–93. 10.1007/s00221-006-0365-2 16489434

[B36] Mochizuki H. Huang Y. Z. Rothwell J. C. (2004). Interhemispheric interaction between human dorsal premotor and contralateral primary motor cortex. *J. Physiol.* 561(Pt 1) 331–338. 10.1113/jphysiol.2004.072843 15459244 PMC1665328

[B37] Morishita T. Ninomiya M. Uehara K. Funase K. (2011). Increased excitability and reduced intracortical inhibition in the ipsilateral primary motor cortex during a fine-motor manipulation task. *Brain Res.* 1371 65–73. 10.1016/j.brainres.2010.11.049 21093420

[B38] Morishita T. Uehara K. Funase K. (2012). Changes in interhemispheric inhibition from active to resting primary motor cortex during a fine-motor manipulation task. *J. Neurophysiol.* 107 3086–3094. 10.1152/jn.00888.2011 22422998

[B39] Muellbacher W. Facchini S. Boroojerdi B. Hallett M. (2000). Changes in motor cortex excitability during ipsilateral hand muscle activation in humans. *Clin. Neurophysiol.* 111 344–349. 10.1016/s1388-2457(99)00243-6 10680571

[B40] Nakamura H. Kitagawa H. Kawaguchi Y. Tsuji H. (1997). Intracortical facilitation and inhibition after transcranial magnetic stimulation in conscious humans. *J. Physiol.* 498(Pt 3) 817–823. 10.1113/jphysiol.1997.sp021905 9051592 PMC1159197

[B41] Netz J. Ziemann U. Homberg V. (1995). Hemispheric asymmetry of transcallosal inhibition in man. *Exp. Brain Res.* 104 527–533. 10.1007/BF00231987 7589304

[B42] Ni Z. Muller-Dahlhaus F. Chen R. Ziemann U. (2011). Triple-pulse TMS to study interactions between neural circuits in human cortex. *Brain Stimul.* 4 281–293. 10.1016/j.brs.2011.01.002 22032744

[B43] Oldfield R. C. (1971). The assessment and analysis of handedness: the edinburgh inventory. *Neuropsychologia* 9 97–113. 10.1016/0028-3932(71)90067-4 5146491

[B44] Quinn L. Miljevic A. Rurak B. K. Marinovic W. Vallence A. M. (2018). Differential plasticity of extensor and flexor motor cortex representations following visuomotor adaptation. *Exp. Brain Res.* 236 2945–2957. 10.1007/s00221-018-5349-5 30088021

[B45] Reid C. S. Serrien D. J. (2014). Primary motor cortex and ipsilateral control: a TMS study. *Neuroscience* 270 20–26. 10.1016/j.neuroscience.2014.04.005 24726982

[B46] Reis J. Swayne O. B. Vandermeeren Y. Camus M. Dimyan M. A. Harris-Love M. (2008). Contribution of transcranial magnetic stimulation to the understanding of cortical mechanisms involved in motor control. *J. Physiol.* 586 325–351. 10.1113/jphysiol.2007.144824 17974592 PMC2375593

[B47] Rossini P. M. Burke D. Chen R. Cohen L. G. Daskalakis Z. Di Iorio R. (2015). Non-invasive electrical and magnetic stimulation of the brain, spinal cord, roots and peripheral nerves: basic principles and procedures for routine clinical and research application. An updated report from an I.F.C.N. Committee. *Clin. Neurophysiol.* 126 1071–1107. 10.1016/j.clinph.2015.02.001 25797650 PMC6350257

[B48] Sanger T. D. Garg R. R. Chen R. (2001). Interactions between two different inhibitory systems in the human motor cortex. *J. Physiol.-Lon.* 530 307–317. 10.1111/j.1469-7793.2001.0307l.x 11208978 PMC2278414

[B49] Shibasaki H. Sadato N. Lyshkow H. Yonekura Y. Honda M. Nagamine T. (1993). Both primary motor cortex and supplementary motor area play an important role in complex finger movement. *Brain* 116(Pt 6) 1387–1398. 10.1093/brain/116.6.1387 8293277

[B50] Smyth C. Summers J. J. Garry M. I. (2010). Differences in motor learning success are associated with differences in M1 excitability. *Hum. Mov. Sci.* 29 618–630. 10.1016/j.humov.2010.02.006 20356643

[B51] Sohn Y. H. Wiltz K. Hallett M. (2002). Effect of volitional inhibition on cortical inhibitory mechanisms. *J. Neurophysiol.* 88 333–338. 10.1152/jn.2002.88.1.333 12091558

[B52] Taniwaki T. Okayama A. Yoshiura T. Togao O. Nakamura Y. Yamasaki T. (2006). Functional network of the basal ganglia and cerebellar motor loops in vivo: different activation patterns between self-initiated and externally triggered movements. *Neuroimage* 31 745–753. 10.1016/j.neuroimage.2005.12.032 16466678

[B53] Tinazzi M. Zanette G. (1998). Modulation of ipsilateral motor cortex in man during unimanual finger movements of different complexities. *Neurosci. Lett.* 244 121–124. 10.1016/S0304-3940(98)00150-5 9593504

[B54] Triggs W. J. Calvanio R. Macdonell R. A. L. Cros D. Chiappa K. H. (1994). Physiological motor asymmetry in human handedness: evidence from transcranial magnetic stimulation. *Brain Res.* 636 270–276. 10.1016/0006-8993(94)91026-x 8012811

[B55] Uehara K. Morishita T. Kubota S. Funase K. (2013a). Change in the ipsilateral motor cortex excitability is independent from a muscle contraction phase during unilateral repetitive isometric contractions. *PLoS One* 8:e55083. 10.1371/journal.pone.0055083 23383063 PMC3561368

[B56] Uehara K. Morishita T. Kubota S. Funase K. (2013b). Neural mechanisms underlying the changes in ipsilateral primary motor cortex excitability during unilateral rhythmic muscle contraction. *Behav. Brain Res.* 240 33–45. 10.1016/j.bbr.2012.10.053 23174210

[B57] Valls-Sole J. Pascual-Leone A. Wassermann E. M. Hallett M. (1992). Human motor evoked responses to paired transcranial magnetic stimuli. *Electroencephalogr. Clin. Neurophysiol.* 85 355–364. 10.1016/0168-5597(92)90048-g 1282453

[B58] van den Berg F. E. Swinnen S. P. Wenderoth N. (2011). Excitability of the motor cortex ipsilateral to the moving body side depends on spatio-temporal task complexity and hemispheric specialization. *PLoS One* 6:e17742. 10.1371/journal.pone.0017742 21408031 PMC3052419

[B59] Verstynen T. Ivry R. B. (2011). Network dynamics mediating ipsilateral motor cortex activity during unimanual actions. *J. Cogn. Neurosci.* 23 2468–2480. 10.1162/jocn.2011.21612 21268666

[B60] Wassermann E. M. Samii A. Mercuri B. Ikoma K. Oddo D. Grill S. E. (1996). Responses to paired transcranial magnetic stimuli in resting, active, and recently activated muscles. *Exp. Brain Res.* 109 158–163. 10.1007/BF00228638 8740220

[B61] Yamada N. Kakuda W. Senoo A. Kondo T. Mitani S. Shimizu M. (2013). Functional cortical reorganization after low-frequency repetitive transcranial magnetic stimulation plus intensive occupational therapy for upper limb hemiparesis: evaluation by functional magnetic resonance imaging in poststroke patients. *Int. J. Stroke* 8 422–429. 10.1111/ijs.12056 23692672

[B62] Ziemann U. Hallett M. (2001). Hemispheric asymmetry of ipsilateral motor cortex activation during unimanual motor tasks: further evidence for motor dominance. *Clin. Neurophysiol.* 112 107–113. 10.1016/S1388-2457(00)00502-2 11137667

[B63] Ziemann U. Lonnecker S. Steinhoff B. J. Paulus W. (1996a). The effect of lorazepam on the motor cortical excitability in man. *Exp. Brain Res.* 109 127–135. 10.1007/BF00228633 8740215

[B64] Ziemann U. Rothwell J. C. Ridding M. C. (1996b). Interaction between intracortical inhibition and facilitation in human motor cortex. *J. Physiol.* 496(Pt 3) 873–881. 10.1113/jphysiol.1996.sp021734 8930851 PMC1160871

